# Is the assumption of equal distances between global assessment categories used in borderline regression valid?

**DOI:** 10.1186/s12909-022-03753-5

**Published:** 2022-10-05

**Authors:** Patrick J. McGown, Celia A. Brown, Ann Sebastian, Ricardo Le, Anjali Amin, Andrew Greenland, Amir H. Sam

**Affiliations:** 1 Imperial College School of Medicine, Imperial College London, London, UK; 2 Warwick Medical School, University of Warwick, Warwick, UK

**Keywords:** Borderline regression, Borderline pass, Standard setting, Clinical examination, Assessment, Pass mark

## Abstract

**Background:**

Standard setting for clinical examinations typically uses the borderline regression method to set the pass mark. An assumption made in using this method is that there are equal intervals between global ratings (GR) (e.g. Fail, Borderline Pass, Clear Pass, Good and Excellent). However, this assumption has never been tested in the medical literature to the best of our knowledge. We examine if the assumption of equal intervals between GR is met, and the potential implications for student outcomes.

**Methods:**

Clinical finals examiners were recruited across two institutions to place the typical ‘Borderline Pass’, ‘Clear Pass’ and ‘Good’ candidate on a continuous slider scale between a typical ‘Fail’ candidate at point 0 and a typical ‘Excellent’ candidate at point 1. Results were analysed using one-sample t-testing of each interval to an equal interval size of 0.25.

Secondary data analysis was performed on summative assessment scores for 94 clinical stations and 1191 medical student examination outcomes in the final 2 years of study at a single centre.

**Results:**

On a scale from 0.00 (Fail) to 1.00 (Excellent), mean examiner GRs for ‘Borderline Pass’, ‘Clear Pass’ and ‘Good’ were 0.33, 0.55 and 0.77 respectively.

All of the four intervals between GRs (Fail-Borderline Pass, Borderline Pass-Clear Pass, Clear Pass-Good, Good-Excellent) were statistically significantly different to the expected value of 0.25 (all *p*-values < 0.0125).

An ordinal linear regression using mean examiner GRs was performed for each of the 94 stations, to determine pass marks out of 24. This increased pass marks for all 94 stations compared with the original GR locations (mean increase 0.21), and caused one additional fail by overall exam pass mark (out of 1191 students) and 92 additional station fails (out of 11,346 stations).

**Conclusions:**

Although the current assumption of equal intervals between GRs across the performance spectrum is not met, and an adjusted regression equation causes an increase in station pass marks, the effect on overall exam pass/fail outcomes is modest.

**Supplementary Information:**

The online version contains supplementary material available at 10.1186/s12909-022-03753-5.

## Background

Medical school examinations are an important barometer of competence required to enable entry into the medical profession, and there is an ethical and patient safety responsibility for medical schools to ensure their testing processes are robust and fair [1]. Indeed, low scores in clinical examinations are associated with increased complaints to medical regulatory authorities for postgraduates [2].

**Fig. 1:**
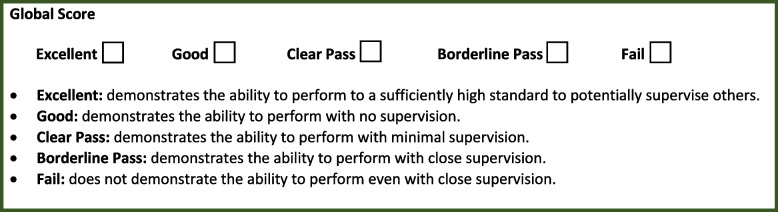
Example of global ratings. Legend: Example of Global Ratings scale used at Imperial College School of Medicine

Examinations based on observations of simulated clinical examinations are widely used in high-stakes clinical assessment [3], and typically involve a sequential assessment of students through structured stations [4]. During the marking process for each station, a candidate is awarded checklist/domain-based marks for specific tasks, which are summed to give a total score for that station. Whilst candidate behaviours are pragmatically converted into numbers for scoring purposes, it has been argued that combining component sub-scores has significant limitations [5]. Examiners also provide a global rating (GR) of the candidate’s performance in that station. A range of categorical scales with various GRs are used at different institutions. These GRs are often made on a 4 or 5-point ordinal scale whereby they are empirically placed in rank order from ‘Fail’ to ‘Excellent’, with interim points on the scale such as ‘Borderline Pass’, ‘Clear Pass’ and ‘Good’. A ‘Borderline Pass’ candidate is often considered as the benchmark of a ‘just-passing’ candidate (Fig. 1).

**Fig. 2:**
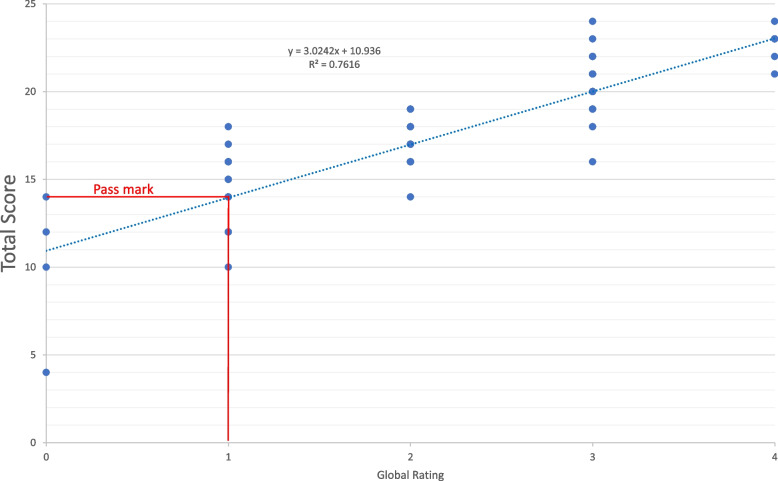
Pass mark calculation by borderline regression

The examination pass mark is typically set using the borderline regression method [6]. This is perceived as the ‘gold-standard’ standard-setting method for high-stakes Objective Structured Clinical Examinations (OSCEs), as there is less variance in the borderline regression approach than alternative standard-setting techniques such as the Borderline-Group or Angoff methods [7]. To set the pass mark for each station, GRs are firstly converted into interval numerical values (Fail = 0, Borderline Pass = 1, Clear Pass = 2, Good = 3, Excellent = 4). Secondly, the total scores awarded to candidates for the station are plotted on the y-axis of a graph against the numerical value for the GR they received for that station on the x-axis. A best-fit line (using ordinal linear regression) is drawn from one end of the scale to the other. The point at which the best-fit line intersects the ‘Borderline Pass’ GR (Fig. 2) provides the pass mark for the station [8].

Previous studies into the borderline regression method for standard setting have assumed that the GRs scale is interval as well as ordinal, with equal intervals between the ordinal GRs along the assessment scale [9]. We performed a PubMed database search using keywords “Borderline Regression” AND “standard setting” OR “global ratings”, and the validity of this assumption has not previously been investigated in the medical literature to the best of our knowledge. However, examiners may differ on what they perceive to be a borderline candidate [10], and therefore they may also differ in how far along the performance scale they place the ‘Borderline Pass’ (and other) categories. If this assumption of equal intervals does not hold, there might be important ramifications for the validity of pass mark setting and the number of students who would pass/fail an examination.

We therefore set out to determine two questions:

- Do examiners of clinical examinations for medical students place the GR locations in an evenly spaced distribution on the performance spectrum?
- What effect does moving the GR locations have on pass/fail outcomes of students sitting examinations which are standard set using the borderline regression method?

## Methods

Clinical finals examiners at Imperial College School of Medicine and Warwick Medical School were recruited via email and at examiner training sessions at both sites. They were provided with a participant information sheet and asked to complete an anonymous online questionnaire on the Qualtrics platform, to place the typical ‘Borderline Pass’, ‘Clear Pass’ and ‘Good’ candidate on a continuous slider scale between a typical ‘Fail’ candidate at point 0 and a typical ‘Excellent’ candidate at point 1 (Additional file 1).

A slider format was chosen to allow for more nuanced data collection than a typical Likert-type scale which is constrained by integers [11], and to permit more precise analysis between items [12] whilst still providing the respondent with the ability to make comparative judgements [13]. Scale labels were omitted to guard against score clustering [14].

The examiner pool who were recruited were all experienced and had undergone examination-specific orientation and training, in keeping with accepted practice to improve scoring accuracy for clinical examinations [15]. Examiners at two institutions were recruited to improve applicability of results to other centres. Both sites used the 5-point GR spectrum used in this study.

The responses were analysed for mean and standard deviation, and one-sample t-testing was performed to test the null hypothesis that each interval was equal to 0.25. The critical *p*-value was set at 0.0125 to account for the use of four tests.

The examiner GR means were used in a secondary data analysis of potential pass/fail outcomes from the clinical finals examination results of students at Imperial College School of Medicine (2019–2021). Results for 94 stations from clinical finals for 1191 students over four exams were included in the analysis (11,346 student-station interactions). For each station, examiner-perceived means of GR locations were used to recalculate the borderline regression equation and pass mark. The pass mark was set at the station score (y-axis) where the adjusted regression line met the adjusted ‘Borderline Pass’ location on the GR spectrum (x-axis).

Two approaches to determining student examination outcomes were analysed: station-level and overall exam-level. At station-level, the number and proportion of students who failed each station using the standard borderline regression pass mark was compared to the number and proportion who failed the same station using the adjusted borderline regression pass mark. At exam-level, the number and proportion of students who failed to meet institutional passing criteria using the standard borderline regression pass marks was compared to the number and proportion of students who failed using adjusted station pass marks.

One-proportion Z-testing was performed to test for differences between station fail rates using the adjusted borderline regression calculation versus the standard. The null hypothesis was that there would be no change to fail rates. The critical *p*-value was set at 0.01 to account for the use of five tests (one test for each of the four examinations studied, and one test for all four examinations combined).

To pass an examination at the institution studied, students must meet the following criteria:

- Overall score (sum of all station scores for the candidate) ≥ overall pass mark (sum of borderline regression pass marks for each station)
- Pass ≥50% of stations
- Overall domain score > overall domain pass mark (for the 4 domains examined within each station (Clinical skills; Formulation of clinical issues; Discussion of management; Professionalism and patient centred approach))

Domain passing scores were not influenced by our intervention and therefore we only investigated change to student pass/fail outcomes based on the first two criteria.

All data were anonymised, and Microsoft Excel was used for analysis.

A sample size calculation for the first research question was undertaken using Stata v17. Using the following parameters, we estimated that 104 responses to the questionnaire would be required to detect a difference in interval size of 0.05 to our hypothesised mean interval of 0.25: alpha = 0.0125, power = 80%, standard deviation of interval sizes across respondents = 0.15.

Ethics approval was received from the Imperial College London Educational Ethics Review Process (EERP2122–020).

## Results

One hundred thirty-two responses were received and 15 were excluded as not valid for not following the defined order of domain categories (Fail - > Borderline Pass - > Clear Pass - > Good - > Excellent) or for placing one or more of the GR slider results at the extremes of the scale (i.e. in the same location as a typical ‘Fail’ or ‘Excellent’ rating).

The results from the remaining 117 responses were included for data analysis (Table 1).

**Table 1:** Examiner perceptions of GR locations across the performance spectrum

Global rating	Examiner Mean	95% Confidence interval of the mean	Standard deviation
Borderline Pass	0.33	0.30–0.35	0.15
Clear Pass	0.55	0.53–0.57	0.11
Good	0.77	0.76–0.79	0.09

*Legend*: Data from 117 examiners. Scale defined as typical ‘Fail’ candidate at 0.00 and typical ‘Excellent’ candidate at 1.00

Data were analysed to calculate the perceived intervals between each pair of adjacent GR locations for each individual examiner, and subsequently mean interval values across examiners were calculated. The examiner perception data were normally distributed, and one sample t-testing was performed on the intervals between GRs to examine the assumption of equal distribution of GRs along the GR spectrum (i.e. that each interval equals 0.25). The results are displayed in Table 2.

**Table 2:** Examiner perceptions of intervals between global scoring domains

GR interval	Mean interval	95% Confidence interval of the GR interval	T-statistic	*P*-value (Critical *p*-value =0.0125)
Fail to Borderline Pass	0.33	0.30–0.35	5.84	< 0.001
Borderline Pass to Clear Pass	0.22	0.20–0.23	4.33	< 0.001
Clear Pass to Good	0.23	0.21–0.24	3.50	< 0.001
Good to Excellent	0.23	0.21–0.24	2.57	0.006

*Legend*: Data from 117 examiners. Null hypothesis that each interval between GRs = 0.25

All of the *p*-values were statistically significant for the intervals between GR locations; therefore we can reject the null hypothesis and conclude that the intervals between GR locations are not equal to 0.25. Thus, the assumption of equal intervals in the borderline regression method is not met.

**Fig. 3:**
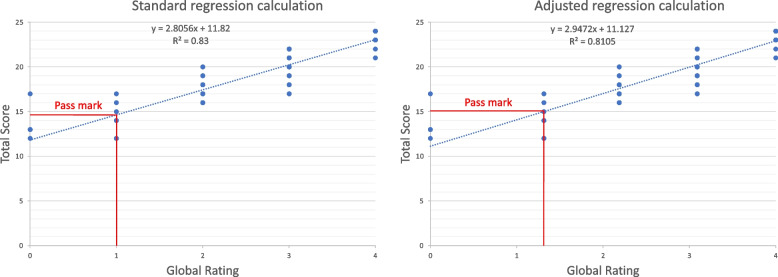
Example of recalculated regression line using adjusted global ratings

Using the examiner-perceived mean GR locations along the performance spectrum, we then recalculated the borderline regression equation (Fig. 3) based on these non-equal GR intervals, for each station in the four most recent finals’ examinations at one institution (94 stations total).

For all stations the r-squared metric remained > 0.5, indicating a good linear correlation between scores and GRs [9]. The effects on student assessment outcomes at station-level and overall exam-level are shown in Table 3.

**Table 3:** Effect of adjusted regression calculation on examination and student outcomes

	Change in exam pass mark (percentage points)	Change in exam-level fails	Stations with increased pass mark	Stations with increased integer pass mark	Change in station fails/total number of student-station interactions	Change in exam-level fails due to criterion of passing ≥50% of stations
Exam 1	+ 0.9	0/310	36/36	7/36	+ 16/3720	0/310
Exam 2	+ 0.7	0/289	10/10	2/10	+ 29/2890	0/289
Exam 3	+ 1.2	0/287	24/24	7/24	+ 12/2296	0/287
Exam 4	+ 1.2	+ 1/305	24/24	11/24	+ 35/2440	0/305
Overall	+ 1.0	+ 1/1191	94/94	37/94	+ 92/11346	0/1191

*Legend*: Data from four summative finals examinations at the institution studied

In each of the 94 stations the pass mark increased using the adjusted borderline regression calculation, with a mean increase of 0.24 marks out of 24 (1%). This led to 92 additional fail grades being awarded at station level (0.01%). The effects of the standard and adjusted regression calculations on fail rates are shown in Table 4.

**Table 4:** Effects of standard and adjusted regression calculations on fail rates

	Station fails using standard regression calculation	Fail rate (standard calculation)	Station fails using adjusted regression calculation	Fail rate (adjusted calculation)	z-statistic	*p*-value (Critical *p*-value = 0.01)
Exam 1	198/3720	0.05	214/3720	0.06	1.17	0.243
Exam 2	118/2890	0.04	147/2890	0.05	2.73	0.006
Exam 3	167/2296	0.07	179/2296	0.08	0.96	0.335
Exam 4	206/2440	0.08	241/2440	0.10	2.44	0.014
Overall	689/11346	0.06	781/11346	0.07	3.62	< 0.001

*Legend*: Data from four summative finals examinations at the institution studied. Null hypothesis that station fail rate using adjusted regression calculation = station fail rate using standard calculation (two-tailed test)

Considering all stations in all exams, we reject the null hypothesis that the station fail rate is the same using the adjusted regression calculation versus the standard, although the effect size is very small (0.01 percentage point). Up to 7.7% of students could be affected (if each new fail was for a different student), but as discussed above this did not have any effect on exam-level pass/fail outcomes using the conjunctive station passing standard at the institution studied.

The mean overall pass mark (calculated as the sum of the station pass marks) also increased by 1 percentage point using the adjusted regression calculation, which equates to a relative increase of 1.75% versus the pass mark using the standard regression calculation. One additional student out of 1191 would have failed the exam based on overall mark, representing a relative increase of 4% in total fails versus the standard regression calculation where there were 25 fails out of the 1191 candidates (2.1%).

Using the institutional conjunctive standard of passing ≥50% of stations to pass the overall exam, there was no change to overall pass/fail outcomes despite the additional 92 station fails. Students who newly failed to meet the ≥50% station passing conjuncture had already failed by virtue of other passing requirements (overall score or domain scores).

## Discussion

Institutions are expected to quality-assure their assessments including standard setting [9, 16]. Yet based on the existing literature, there is no evidence to support the assumption of equal intervals between GR locations used in the borderline regression method.

The finding that examiners do not perceive intervals between GR locations are equal to 0.25 is important, because this implies that the gold-standard method for standard setting in clinical examinations uses an assumption which is not true. Consequently, there are potential ethical and patient safety ramifications if this significantly affects medical student pass/fail outcomes.

**Fig. 4:**
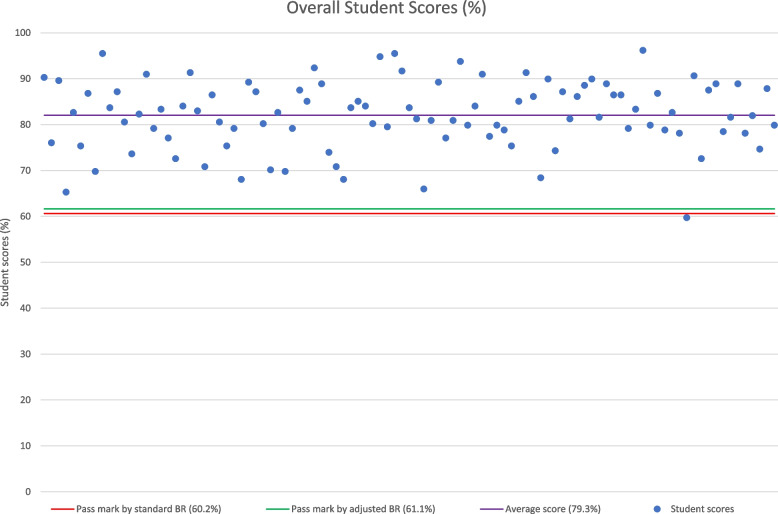
Overall student scores in relation to pass mark and average mark

Indeed, the adjusted regression calculation based on the results of our survey of 120 examiners increased station pass marks and also increased station fail rates versus the standard regression calculation. However, reassuringly the impact on overall pass/fail student outcomes at the institution studied is limited. An average increase in the overall exam pass mark by 1 percentage point does not affect many students, as relatively few students’ scores are clustered around the exam pass mark (Fig. 4).

**Fig. 5:**
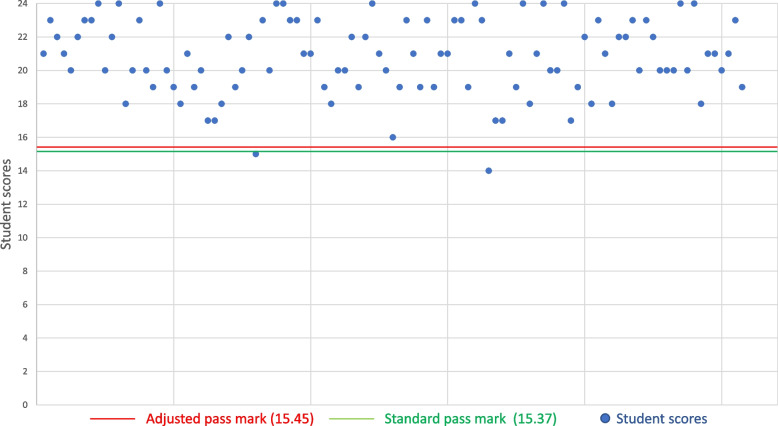
Increased pass mark with adjusted regression calculation but no integer change

Station-level pass/fail differences are important at exam-level in institutions where a station passing conjunctive standard is part of the overall passing criteria [17]. Whilst the station-level pass mark increased across all 94 stations, the mean increase in station-level pass mark (0.24 out of 24 possible marks) was less than one. This is important because student scores are awarded as integer values out of 24 for each station, and students cannot be awarded partial marks, so unless the pass mark increase crosses an integer boundary it does not exert an effect on student outcomes. For example, Fig. 5 shows student scores in a station where the adjusted regression calculation leads to an increased pass mark from 15.37/24 to 15.45/24. Here, the pass mark increase does not cross an integer value and thus there are no additional fails; any student who scored 15/24 will fail whilst any student who scored 16/24 will pass using both the standard and the adjusted regression calculations.

**Fig. 6:**
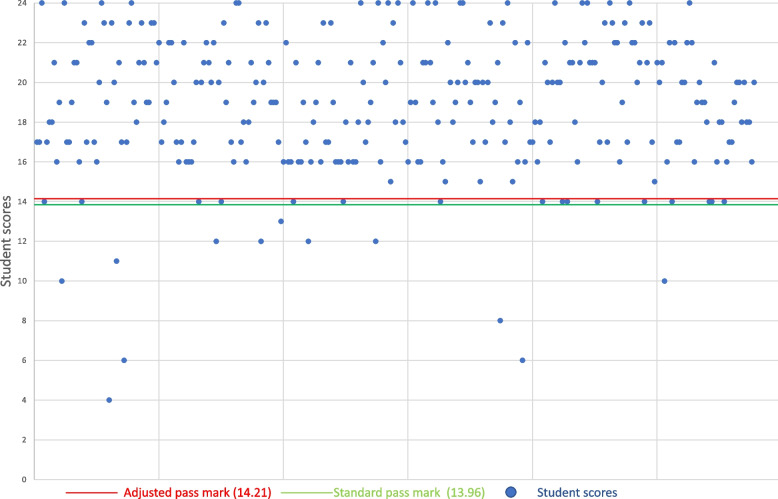
Change in integer pass mark using adjusted regression calculation versus standard

Conversely, Fig. 6 shows an instance where the increased pass mark crosses an integer value from 13.96/24 to 14.21/24. Students who scored 14/24 would pass the station based on the standard regression equation, but would fail based on the adjusted regression equation; this would lead to an additional 6 fail scores for the station.

The need for an integer change in the pass mark for any impact on the pass rate reduces the impact of increased station pass marks on student outcomes using the adjusted regression equation. However, there was still an integer change in pass mark in 37/94 stations (39%). This caused 92 additional station fails versus the standard regression equation, and although there was a statistically significant difference between station fail rates using the adjusted regression calculation versus the standard, the effect size was very small (0.01 percentage point). This may reflect a large sample size causing a tiny effect to be statistically significant.

The effect of additional station fails on exam-level outcomes depends on the passing criteria used at each institution. The use of conjunctive standards such as minimum station passing requirements as an addition to an overall cut score is acknowledged to cause more fails at overall exam-level [3], and institutions employ different stringencies of conjunctive standards in their assessments [18]. Using our institution’s conjunctive standard of passing ≥50% stations, the additional station fails with adjusted GRs did not impact upon overall exam-level pass/fail outcomes. All students who would fail to pass ≥50% of stations with the adjusted regression calculation had already failed based on other passing criteria (overall or domain score requirements). This indicates that despite differences in examiner perceptions of GR locations across the performance spectrum, the conventional borderline regression method is reassuringly robust and valid at a ≥ 50% station passing conjunctive.

**Fig. 7:**
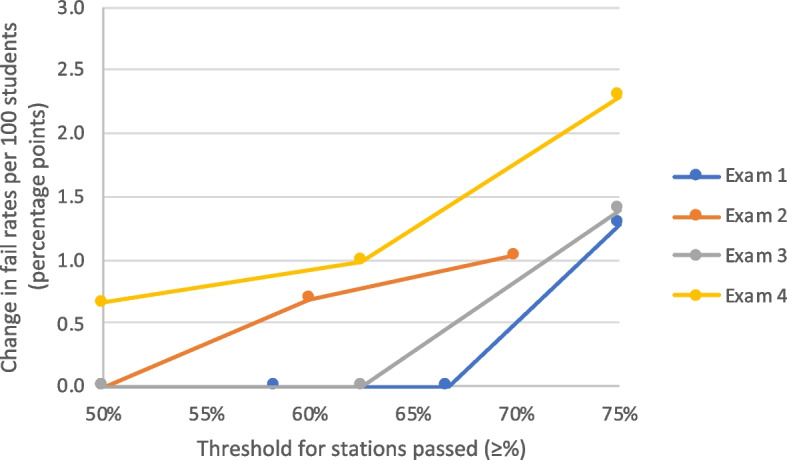
Absolute change in exam-level fail rates by station passing requirements (adjusted regression calculation versus standard). Legend: Change in exam-level fail rates per 100 students (absolute rate change in percentage points) by station passing requirements at different thresholds, when using adjusted borderline regression calculations versus standard calculations. Data from Exam 1 (12 stations), Exam 2 (10 stations), Exam 3 (8 stations) and Exam 4 (10 stations). Datapoints shown for station passing thresholds equate to ≥4/8, ≥5/10 or ≥ 6/12 stations (≥50%), ≥7/12 stations (≥58.3%), ≥6/10 stations (≥60%), ≥5/8 stations (≥62.5%), ≥8/12 stations (≥66.6%), ≥7/10 stations (≥70%) and ≥ 6/8 or ≥ 9/12 stations (≥75%)

Understandably, if more stringent conjunctive station pass requirements were used, both the standard and adjusted regression calculation would lead to additional overall fails. However, as the number of stations required to pass increases, the adjusted regression calculation leads to higher numbers of additional overall exam fails by station passing conjunctive compared to the standard calculation (Fig. 7).

There are some limitations to this study. Examiner perception data were derived from only two UK institutions and these may likely differ from others in their examiner training content and usual standards. Additionally, although multiple examination cohorts were used for secondary data analysis to improve reliability, the student examination data analysed comes from a single centre and thus the effect on pass/fail outcomes may not be transferable to other institutions. Furthermore, the examination data used for secondary analysis may not be without bias; examiners may use varied frames of reference [19], and examiner variance can lead to scoring inconsistencies even when assessing the same encounter [20]. Finally, this study was conducted on the basis of using a 5-point rating scale for GRs. Examiner perceptions of GR locations along the performance spectrum may differ where 4-point or 6-point scales are used, or at institutions with differing GR descriptors, and this might not have the same impact upon the borderline regression calculation.

## Conclusion

In conclusion, our results challenge the assumption of equal intervals between GR domains in the borderline regression method. An adjusted regression calculation would lead to increased pass marks at station and exam-level, and result in lower pass rates. However, the effect size is not large, and there would be a very small impact on pass/fail outcomes at an overall exam-level using our institutional station pass requirements of passing ≥50% of stations. This effect would vary by institution based on passing criteria, and increases if station pass requirements are more stringent.

## Supplementary Information

**Additional file 1.**

## Data Availability

The secondary data that support the findings of this study are available from Imperial College London but restrictions apply to the availability of the institutional assessment data, which were used under license for the current study, and so are not publicly available. The dataset generated and analysed during the current study pertaining to examiner perceptions of global rating scores is available in the Mendeley repository, DOI: 10.17632/nznbxmbh99.2

## Abbreviations

GR: Global Rating
OSCEs: Objective Structured Clinical Examinations
