# Supplementary figures and images for: Is the assumption of equal distances between global assessment categories used in borderline regression valid?
Source: BMC Med Educ. 2022 Oct 5;22:708. doi: 10.1186/s12909-022-03753-5 (PMC9536020; doi:10.1186/s12909-022-03753-5)

ADDITIONAL FILE 1:

Additional file 1: Qualtrics Slider Questionnaire for examiners


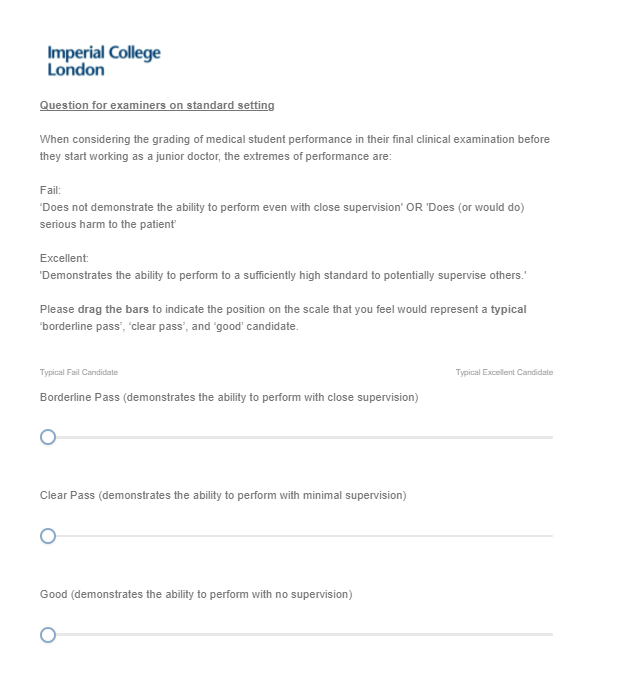

Supplement: Supplementary file 1 — Additional file 1. [file 12909_2022_3753_MOESM1_ESM.docx]
